# Pain Phenotypes, Treatment Patterns, and Utilization Burden Among Patients with Inflammatory Bowel Disease Referred to a Tertiary Pain Clinic: A Retrospective Cohort Study

**DOI:** 10.3390/biomedicines14071422

**Published:** 2026-06-23

**Authors:** Shachar Zion Shemesh, Paz Kelmer, Bella Ungar, Yotam Hadari, Lior Ungar

**Affiliations:** 1 Department of Neurosurgery, Sheba Medical Center, Ramat Gan 6997801, Israel; 2 Gray Faculty of Medical and Health Sciences, Tel Aviv University, Tel Aviv 6997801, Israel; 3 Gastroenterology Institute, Sheba Medical Center, Tel Hashomer, Ramat Gan 6997801, Israel; 4 Pain Center, Sheba Medical Center, Ramat Gan 6997801, Israel; 5 Dina Recanati School of Medicine, Reichman University, Herzliya 4610101, Israel

**Keywords:** inflammatory bowel disease, Crohn’s disease, ulcerative colitis, chronic pain, pain clinic, musculoskeletal pain, neuropathic pain, interventional pain, retrospective cohort

## Abstract

**Background:** Pain is a prominent and disabling manifestation of inflammatory bowel disease (IBD), including abdominal, pelvic, musculoskeletal, axial, and neuropathic pain phenotypes. Patients referred to pain clinics represent a selected subgroup with clinically meaningful, persistent, refractory, or diagnostically complex pain. **Objective:** To characterize pain phenotypes, treatment patterns, interventional pain-care exposure, and utilization burden among patients with IBD evaluated in tertiary pain-clinic settings and to explore differences between Crohn’s disease and ulcerative colitis patients. **Methods:** We performed a retrospective electronic medical-record cohort study of patients with documented IBD who were evaluated in pain-clinic settings between 24 October 2010 and 14 May 2026. Repeated clinical entries were aggregated into unique visit dates and patient-level variables. IBD diagnosis, pain phenotypes, treatment documentation, interventional procedures, and pain-clinic utilization were summarized descriptively using counts, percentages, means, medians, interquartile ranges, and ranges as appropriate. Crohn’s disease and ulcerative colitis subgroups were compared using univariable odds ratios with 95% confidence intervals and two-sided *p*-values. Because repeated clinical entries could belong to the same patient, the primary analytic unit was the patient rather than the individual note. **Results:** The source dataset included 19,615 clinical entries representing 7053 unique pain-clinic visits among 596 unique patients with IBD. The cohort included 314 patients with Crohn’s disease (52.7%), 247 with ulcerative colitis (41.4%), and 35 with IBD-unclassified (5.9%). The mean number of pain-clinic visits per patient was 11.8, with a median of four visits (interquartile range, 1–11). The dominant patient-level pain phenotypes were limb or peripheral joint pain (395/596, 66.3%), back or axial spine pain (358/596, 60.1%), and abdominal or pelvic pain (246/596, 41.3%). Overall, 437 patients (73.3%) had documentation of at least one interventional pain procedure. Compared with ulcerative colitis, Crohn’s disease was associated with higher documentation of abdominal or pelvic pain (148/314, 47.1% vs. 82/247, 33.2%; odds ratio, 1.79; 95% confidence interval, 1.27–2.53; *p* = 0.001) and fibromyalgia-like or widespread pain (83/314, 26.4% vs. 39/247, 15.8%; odds ratio, 1.92; 95% confidence interval, 1.25–2.93; *p* = 0.0027). In contrast, radiofrequency procedures (59/314, 18.8% vs. 78/247, 31.6%; odds ratio, 0.50; 95% confidence interval, 0.34–0.74; *p* = 0.0005) and facet or medial branch procedures (79/314, 25.2% vs. 87/247, 35.2%; odds ratio, 0.62; 95% confidence interval, 0.43–0.89; *p* = 0.012) were less frequently documented in Crohn’s disease than in ulcerative colitis. **Conclusions:** Among patients with IBD referred to tertiary pain-clinic evaluation, pain was heterogeneous and predominantly musculoskeletal, axial, neuropathic, and procedurally targetable rather than exclusively visceral. These findings support structured, mechanism-based pain assessment integrated with gastroenterology, rheumatology, and pain-medicine care.

## 1. Introduction

Inflammatory bowel disease (IBD), comprising Crohn’s disease and ulcerative colitis, is a chronic, relapsing, immune-mediated disorder of the gastrointestinal tract with a substantial and increasing global burden. The Global Burden of Disease 2017 analysis estimated approximately 6.8 million prevalent cases of IBD worldwide, and contemporary epidemiologic data indicate that IBD is no longer confined to historically high-incidence regions in North America, Europe, and Oceania [1]. Rather, IBD has become a global disease, with increasing incidence in newly industrialized and emerging regions and accumulating prevalence in regions where incidence has plateaued or declined [2]. This evolving epidemiologic burden is clinically important because IBD is associated not only with intestinal inflammation, hospitalization, surgery, and advanced medical therapy but also with chronic symptoms that may persist despite control of inflammatory activity.

The pathophysiology of IBD is multifactorial and incompletely understood. Current models emphasize interactions among genetic susceptibility, epithelial barrier dysfunction, intestinal microbial dysbiosis, environmental exposures, and dysregulated mucosal immune responses [3,4]. Crohn’s disease may affect any segment of the gastrointestinal tract and can be complicated by stricturing, penetrating, fistulizing, perianal, and postoperative disease, whereas ulcerative colitis is characterized by continuous mucosal inflammation of the colon extending proximally from the rectum [3,4]. Although intestinal inflammation defines IBD, the clinical manifestations of the disease frequently extend beyond the bowel. Extraintestinal manifestations are common and may involve the musculoskeletal, dermatologic, ocular, hepatobiliary, vascular, and hematologic systems; contemporary European Crohn’s and Colitis Organisation guidance notes that up to 50% of patients with IBD may develop at least one extraintestinal manifestation [5]. Musculoskeletal manifestations, including peripheral arthropathy, enthesitis, sacroiliitis, and axial spondyloarthritis, are particularly relevant to pain medicine because they may produce limb, joint, buttock, neck, and back pain [5,6].

Modern IBD management has shifted toward objective treat-to-target strategies that integrate symptom assessment with biomarkers, endoscopy, cross-sectional imaging, and individualized therapeutic selection. Current treatment options include 5-aminosalicylates for selected ulcerative colitis patients, corticosteroids for induction of remission but not long-term maintenance, immunomodulators, biologic therapies targeting tumor necrosis factor, integrins, interleukin-12/23 or interleukin-23 pathways, and small molecules such as Janus kinase inhibitors and sphingosine-1-phosphate receptor modulators [7,8,9,10]. Surgery remains an important component of care for medically refractory disease, stricturing or penetrating complications, dysplasia, cancer prevention, and selected disease phenotypes [7,8,9,10]. However, successful control of luminal inflammation does not necessarily eliminate pain, and persistent pain may reflect mechanisms that extend beyond active bowel inflammation alone.

Crohn’s disease patients had higher documentation of abdominal or pelvic pain and fibromyalgia-like or widespread pain. This pattern is consistent with the transmural, segmental, stricturing, fistulizing, perianal, and postoperative complexity of Crohn’s disease, but it should not be interpreted as proof of a single disease-specific pain mechanism. Recent mechanistic studies emphasize the heterogeneity of IBD biology, including immune-signaling pathways such as TLR4/NF-κB activation and microbiome-related susceptibility to complications such as Clostridioides difficile infection in Crohn’s disease [10,11,12,13,14,15]. These data support the broader concept that Crohn’s disease may involve inflammatory, microbial, metabolic, structural, and postoperative contributors to symptom burden. However, they do not directly establish the mechanism of chronic pain in our cohort. Conversely, the higher documentation of radiofrequency and facet or medial branch procedures among ulcerative colitis patients may reflect referral patterns, age, degenerative spine disease, local practice patterns, or unmeasured rheumatologic comorbidity rather than a true ulcerative-colitis-specific axial pain phenotype. These discrepancies highlight the need for prospective phenotyping that integrates gastroenterologic activity, rheumatologic assessment, spine imaging, pain-mechanism classification, and longitudinal treatment response.

Pain is among the most common and disabling symptoms experienced by patients with IBD and is associated with impaired quality of life, psychological distress, sleep disturbance, functional limitation, and increased healthcare use [11,12,13]. Abdominal or pelvic pain may arise from active inflammation, strictures, fistulas, abscesses, adhesions, postoperative changes, visceral hypersensitivity, irritable bowel syndrome-like overlap, pelvic floor dysfunction, or centrally mediated pain mechanisms [11,16,17,18]. Importantly, pain may persist even during biochemical, endoscopic, or radiologic remission. Systematic reviews have shown that irritable bowel syndrome-type symptoms are frequent among patients with IBD, including those in remission, supporting the concept that persistent symptoms may reflect brain–gut axis dysfunction, visceral hypersensitivity, and non-inflammatory mechanisms rather than ongoing mucosal inflammation alone [16,17]. Recent guidance from the American Gastroenterological Association emphasizes that chronic abdominal pain in IBD should be evaluated using a multidimensional framework and that multidisciplinary care, including behavioral therapies and neuromodulators when appropriate, may be required when central mechanisms contribute to pain persistence [18].

In addition to visceral and functional pain, patients with IBD may experience musculoskeletal, neuropathic, postsurgical, myofascial, pelvic, and nociplastic pain phenotypes. Neuropathic and centrally amplified pain states are clinically important because they are not expected to respond adequately to escalation of anti-inflammatory therapy alone. Conversely, attributing all persistent pain in IBD to functional or centralized mechanisms may delay recognition of active inflammation, structural complications, or inflammatory musculoskeletal disease. Therefore, distinguishing inflammatory visceral pain from axial spine pain, peripheral joint pain, neuropathic pain, postsurgical pain, medication-related pain, opioid-related complications, and nociplastic or widespread pain is a major clinical challenge [18,19,20].

Most previous studies of pain in IBD have been conducted in gastroenterology clinics, population-based cohorts, or disease-specific registries. These studies are essential for estimating symptom prevalence and identifying disease-related correlates, but they do not characterize the selected subgroup of patients with IBD who are referred to tertiary pain services. Referral to a pain clinic implies a distinct clinical population: patients are typically referred because pain is persistent, functionally limiting, refractory to first-line treatment, diagnostically complex, or suggestive of non-visceral pain generators. As a result, the pain-clinic IBD population may differ substantially from the general IBD population in pain mechanisms, treatment exposure, interventional procedure use, and healthcare utilization. This subgroup remains insufficiently described in the literature.

The present study evaluated a large retrospective cohort of patients with IBD referred to tertiary pain-clinic settings over more than 15 years. The primary objective was to characterize patient-level pain phenotypes, medication and treatment documentation, interventional pain-care exposure, and pain-clinic utilization burden. A secondary objective was to explore differences between Crohn’s disease and ulcerative colitis in pain phenotypes and selected interventional procedure patterns. We hypothesized that patients with IBD referred to tertiary pain care would demonstrate a heterogeneous, mechanism-spanning pain phenotype rather than a predominantly visceral abdominal-pain phenotype alone.

## 2. Methods

### 2.1. Study Design

We conducted a retrospective electronic medical-record cohort study of patients with IBD evaluated in tertiary pain-clinic settings. The source dataset contained structured visit metadata and clinical documentation from pain-related encounters between 24 October 2010 and 14 May 2026. The study was designed to describe a specialty referral cohort and was not intended to estimate the prevalence of pain among all patients with IBD. The data were obtained from the electronic medical records of the tertiary Pain Center at Sheba Medical Center, Ramat Gan, Israel.

### 2.2. Patient Population

**Figure 1:**
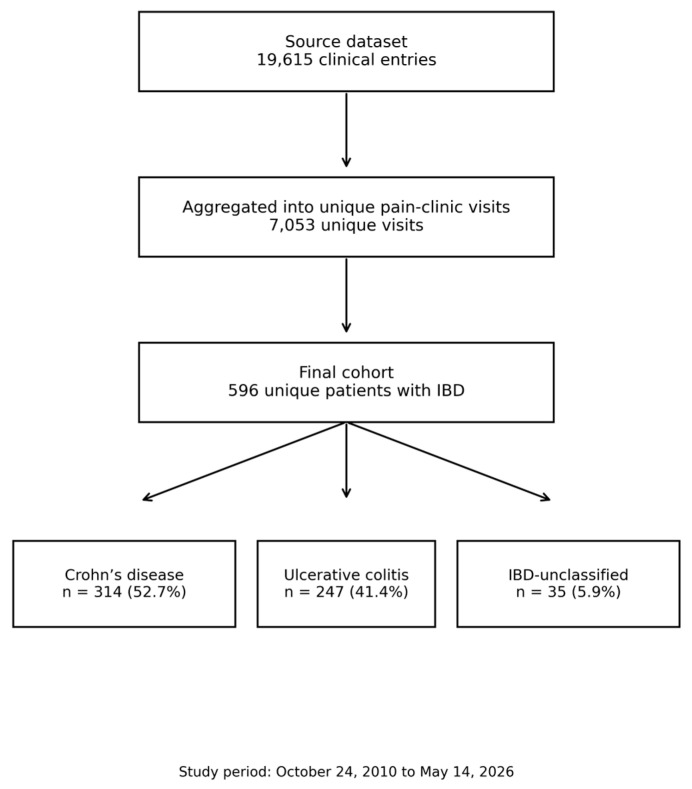
Cohort flow diagram. The source dataset included 19,615 clinical entries. These were aggregated into 7053 unique pain-clinic visits and 596 unique patients with IBD, including 314 with Crohn’s disease, 247 with ulcerative colitis, and 35 with IBD-unclassified.

The analytic cohort included patients with clinical documentation consistent with Crohn’s disease, ulcerative colitis, or IBD-unclassified who had at least one pain-clinic-related encounter during the study period. The source dataset comprised 19,615 clinical entries. Because several entries could correspond to a single clinical visit, unique visits were defined by patient identifier, medical-record identifier, and visit date. Patient-level features were then generated by collapsing repeated entries and visits belonging to the same patient, so that each patient contributed once to each binary clinical feature (Table 1, Figure 1).

### 2.3. Variables and Outcomes

Clinical variables were summarized at both the visit level and the patient level. The principal patient-level variables were IBD diagnosis, number of unique pain-clinic visit dates, pain phenotypes, interventional pain procedures, and medication or treatment-class documentation. Pain phenotypes were defined by systematic review and abstraction of clinical documentation, including presenting complaints, visit summaries, and pain-related clinical descriptions. Categories included limb or peripheral joint pain, back or axial spine pain, abdominal or pelvic pain, neuropathic or complex regional pain syndrome-like features, neck pain, head or craniofacial pain, and fibromyalgia-like or widespread pain. Because the records were generated in real-world clinical practice, categories were not mutually exclusive; a patient could contribute to more than one phenotype if multiple pain syndromes were documented across visits. Pain-clinic utilization was defined as the number of unique pain-clinic visit dates per patient and the proportions of patients with at least 2, at least 5, and at least 10 unique pain-clinic visit dates. Interventional pain-care exposure was defined as documentation of at least one interventional pain procedure category during the study period. These measures reflect pain-clinic contact and procedure documentation, not all-cause healthcare utilization.

### 2.4. Statistical Analysis

Categorical variables are reported as n/N (%). Continuous and count variables are reported as the mean, median, interquartile range (IQR), and range, as appropriate. Because multiple clinical entries and multiple visits could belong to the same patient, the primary unit of analysis was the patient. Repeated entries from the same patient were collapsed into patient-level binary variables, such that each patient contributed only once to each pain phenotype, medication class, or procedure category. Visit-level phenotype frequencies are reported separately as descriptive encounter-level measures and are not used as independent observations for patient-level statistical comparisons. Crohn’s disease and ulcerative colitis subgroups are compared using univariable odds ratios with 95% confidence intervals and two-sided *p*-values for patient-level binary outcomes. The IBD-unclassified group was retained for descriptive cohort reporting but was not used as the primary comparator in Crohn’s disease versus ulcerative colitis analyses. When comparing visit burden between groups, non-normally distributed count data should be analyzed using the Wilcoxon rank-sum test. Because key demographic, rheumatologic, disease-activity, imaging, and biomarker variables were not available in the analytic dataset, inferential comparisons were interpreted as exploratory and hypothesis-generating rather than causal.

## 3. Results

### 3.1. Cohort Overview

The source dataset included 19,615 clinical entries corresponding to 7053 unique pain-clinic visits among 596 patients with IBD. The study period extended from 24 October 2010 through 14 May 2026. Crohn’s disease was documented in 314 patients (52.7%), ulcerative colitis in 247 (41.4%), and IBD-unclassified in 35 (5.9%). Patients with Crohn’s disease accounted for 3606 visits (51.1%), patients with ulcerative colitis accounted for 2947 visits (41.8%), and patients with IBD-unclassified accounted for 500 visits (7.1%). Pain-clinic utilization was skewed. The mean number of visits per patient was 11.8, whereas the median was 4 (interquartile range, 1–11). Recurrent utilization was common: 434 patients (72.8%) had at least two visits, 264 (44.3%) had at least five visits, and 165 (27.7%) had at least ten visits. Thus, the cohort was enriched for patients requiring repeated pain-care contact rather than isolated consultation alone.

### 3.2. Pain Phenotypes

**Figure 2:**
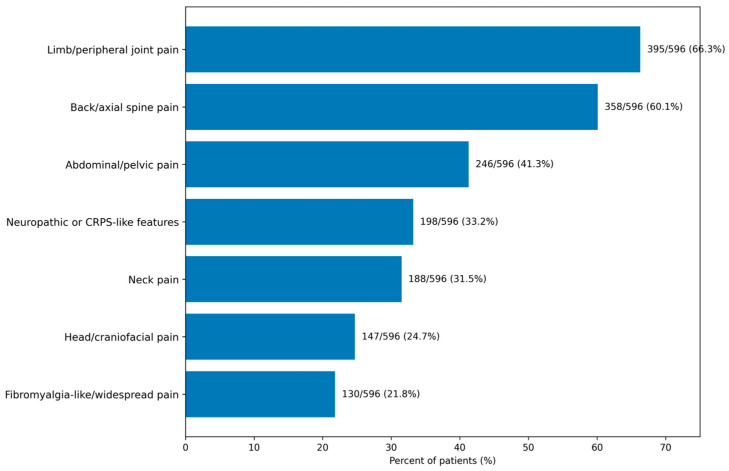
Distribution of patient-level pain phenotypes among IBD patients referred to pain clinics. Bar graph showing limb/peripheral joint pain, back/axial spine pain, abdominal/pelvic pain, neuropathic or CRPS-like features, neck pain, head/craniofacial pain, and fibromyalgia-like/widespread pain.

Pain documentation demonstrated a broad and multidimensional phenotype (Table 2 and Table 3). At the patient level, limb or peripheral joint pain was the most common phenotype, documented in 395 patients (66.3%), followed by back or axial spine pain in 358 patients (60.1%) and abdominal or pelvic pain in 246 patients (41.3%). Neuropathic or complex regional pain syndrome-like features were documented in 198 patients (33.2%), neck pain in 188 (31.5%), head or craniofacial pain in 147 (24.7%), and fibromyalgia-like or widespread pain in 130 (21.8%). Because many patients had multiple encounters, visit-level documentation was analyzed separately and is presented as an encounter-level descriptive measure rather than as an independent patient-level outcome (Table 4). At the visit level, back or axial spine pain and limb or peripheral joint pain were the most frequent categories, documented in 45.9% and 44.3% of visits, respectively. Abdominal or pelvic pain was documented in 22.0% of visits. These findings indicate that even in a cohort defined by IBD, the pain-clinic presentation was not primarily limited to abdominal or pelvic pain (Table 2, Table 3 and Table 4, Figure 2).

### 3.3. Interventional Pain-Care Exposure

Procedural treatment was common. Overall, 437 patients (73.3%) underwent at least one interventional pain procedure. The most frequent procedures were epidural or nerve-root blocks in 181 patients (30.4%), facet or medial branch procedures in 171 (28.7%), and radiofrequency procedures in 145 (24.3%). Additional procedure categories are shown in Table 5. These findings demonstrate substantial interventional pain-care exposure in this referral cohort.

### 3.4. Pain Medications

Medication-class documentation is summarized in Table 6. Gabapentinoids were documented in 176 patients (29.5%), and serotonin-norepinephrine reuptake inhibitor or tricyclic antidepressant therapy was documented in 146 patients (24.5%). Opioid documentation was present in 68 patients (11.4%), and systemic steroid documentation was present in 231 patients (38.8%). Because medication exposure was extracted from clinical documentation rather than pharmacy-dispensing records, these variables should be interpreted as documented medication-class exposure rather than complete medication prevalence or confirmed treatment indication (Table 6).

### 3.5. Crohn’s Disease Versus Ulcerative Colitis

Exploratory patient-level comparisons showed several differences between Crohn’s disease and ulcerative colitis (Table 7). Abdominal or pelvic pain was more frequently documented in Crohn’s disease than in ulcerative colitis (148/314, 47.1% vs. 82/247, 33.2%; odds ratio, 1.79; 95% confidence interval, 1.27–2.53; *p* = 0.001). Fibromyalgia-like or widespread pain was also more frequent in Crohn’s disease (83/314, 26.4% vs. 39/247, 15.8%; odds ratio, 1.92; 95% confidence interval, 1.25–2.93; *p* = 0.0027).

In contrast, ulcerative colitis was associated with more frequent documentation of spine-directed interventional procedures. Radiofrequency procedures were documented in 59 patients with Crohn’s disease (18.8%) and 78 patients with ulcerative colitis (31.6%), corresponding to an odds ratio of 0.50 for Crohn’s disease relative to ulcerative colitis (95% confidence interval, 0.34–0.74; *p* = 0.0005). Facet or medial branch procedures were documented in 79 patients with Crohn’s disease (25.2%) and 87 patients with ulcerative colitis (35.2%), corresponding to an odds ratio of 0.62 for Crohn’s disease relative to ulcerative colitis (95% confidence interval, 0.43–0.89; *p* = 0.012).

**Figure 3:**
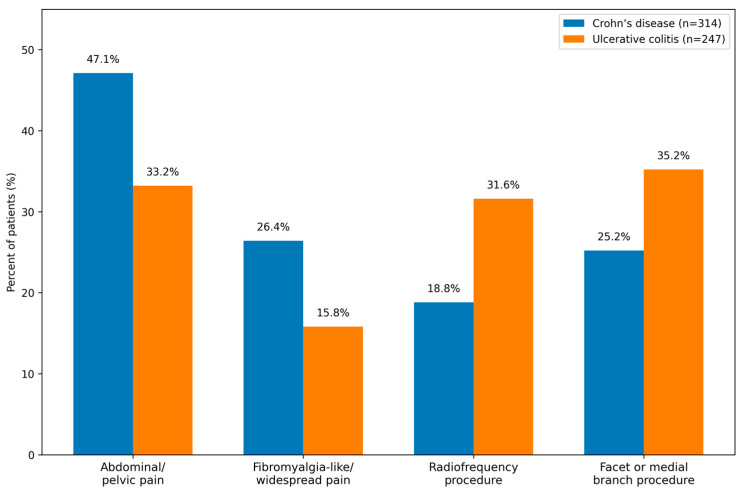
Crohn’s disease versus ulcerative colitis exploratory comparisons. Bar graph comparing abdominal/pelvic pain, fibromyalgia-like/widespread pain, radiofrequency procedures, and facet or medial branch procedures between Crohn’s disease and ulcerative colitis.

Thus, Crohn’s disease was associated with higher documentation of abdominopelvic and widespread pain phenotypes, whereas ulcerative colitis was associated with greater documentation of selected spine-directed procedures. The latter finding should be interpreted as a procedural pattern rather than definitive evidence of a higher prevalence of axial spine disease in ulcerative colitis (Table 7, Figure 3).

## 4. Discussion

This retrospective cohort study describes a large specialty referral population of patients with IBD evaluated in tertiary pain-clinic settings. The principal finding is that the pain phenotype of referred IBD patients is heterogeneous and heavily enriched for musculoskeletal, axial, and neuropathic pain. Although abdominal or pelvic pain was common, it was not the dominant clinical category. Limb or peripheral joint pain and back or axial spine pain were more frequently documented. These results should not be interpreted as prevalence estimates for pain among all patients with IBD. Rather, they define the phenotype of patients with IBD whose pain was severe, persistent, refractory, complex, or focal enough to generate pain-clinic referral.

The predominance of limb, joint, axial spine, and neck pain is clinically plausible and consistent with the recognized musculoskeletal spectrum of IBD. Extraintestinal manifestations of IBD include peripheral arthropathy, enthesitis, and axial spondyloarthritis, and these conditions may produce limb, joint, back, buttock, and neck pain. In addition, patients with chronic inflammatory disease may have coexisting degenerative spine disease, myofascial pain, postoperative pain, deconditioning-related pain, and overlapping neuropathic or nociplastic syndromes. These mechanisms cannot be distinguished definitively in the present dataset. Therefore, the high frequency of axial and peripheral pain in this cohort should be interpreted as a signal that such patients require structured musculoskeletal and rheumatologic evaluation rather than as proof that all axial pain was directly caused by IBD [17,18,19,20,21,22].

Nearly three-quarters of patients had documentation of at least one interventional pain procedure, and the most common procedures were epidural or nerve-root blocks, facet or medial branch procedures, and radiofrequency procedures. These patterns suggest that many patients were treated according to axial spine, radicular, facet-mediated, myofascial, or peripheral nerve pain paradigms. This does not demonstrate procedural effectiveness, nor does it prove that pain was unrelated to IBD. It does, however, show that the clinical pathway for many referred patients extended well beyond gastroenterologic disease control.

Importantly, procedure documentation should not be interpreted as evidence of treatment response, procedural appropriateness, or causal pain mechanism, because standardized pre- and post-procedure pain outcomes were not available.

The relatively frequent documentation of gabapentinoids and serotonin-norepinephrine reuptake inhibitor or tricyclic antidepressant therapy is consistent with clinical recognition of neuropathic or centrally amplified pain features in a meaningful subset of patients. These medication classes are commonly used in neuropathic and centralized pain states, although the present dataset cannot confirm indication, dose, adherence, or response. One-third of the cohort had documented neuropathic or complex regional pain syndrome-like features, and more than one-fifth had fibromyalgia-like or widespread pain. These features are important because chronic pain in IBD may persist after control of inflammatory activity and may require multimodal treatment strategies. A practical clinical framework should, therefore, distinguish inflammatory visceral pain from structural axial pain, peripheral joint pain, neuropathic pain, postsurgical pain, pelvic pain, opioid-related complications, and nociplastic or centralized pain [22,23,24,25].

Crohn’s disease patients had higher documentation of abdominal or pelvic pain and fibromyalgia-like or widespread pain. This may reflect the transmural, segmental, and often surgically complex nature of Crohn’s disease, including stricturing, fistulizing, postoperative, or adhesion-related pain pathways. It may also reflect longer disease burden or centrally amplified pain in some patients. In contrast, ulcerative colitis patients more frequently had documentation of radiofrequency and facet or medial branch procedures. This should be interpreted cautiously as a procedural association rather than a definitive disease-specific pain mechanism [25,26,27,28,29].

A major limitation of this comparison is the absence of adjustment for age, sex, disease duration, rheumatologic diagnoses, degenerative spine disease, imaging findings, prior surgery, inflammatory activity, and medication exposure. These variables may differ between Crohn’s disease and ulcerative colitis and may partly explain the observed procedural differences. In particular, axial spondyloarthritis and other rheumatologic comorbidities are clinically relevant potential confounders in any analysis of back pain, facet procedures, and radiofrequency procedures among patients with IBD. Therefore, the Crohn’s disease versus ulcerative colitis comparisons should be viewed as exploratory and hypothesis-generating [28,29,30,31,32,33].

The pattern of pain-clinic follow-up demonstrated frequent recurrent utilization. The median patient had four unique pain-clinic visit dates, but the distribution was strongly skewed, and approximately 28% of patients had at least ten unique pain-clinic visit dates. Each unique pain-clinic date was counted as a visit, including both evaluation visits and procedural encounters. Therefore, visit burden reflects overall pain-clinic contact rather than physician consultation alone.

Because the present cohort did not include a matched non-IBD pain-clinic comparison group or a general IBD denominator, we cannot determine whether utilization was higher than expected compared with the general pain-clinic population or all patients with IBD. Nevertheless, within this referred IBD cohort, a substantial subgroup demonstrated chronic and complex pain-care needs. Future studies should include matched non-IBD pain-clinic controls and identify predictors of high utilization, including age, sex, disease duration, prior surgery, inflammatory activity, rheumatologic comorbidity, psychiatric comorbidity, opioid exposure, spine disease, socioeconomic factors, and response to specific interventions.

From a care-delivery standpoint, the findings support closer integration between gastroenterology, rheumatology, and pain medicine. We propose a structured referral algorithm for IBD patients with persistent pain. First, clinicians should assess whether pain is accompanied by features of active intestinal inflammation, including change in bowel symptoms, bleeding, fever, weight loss, elevated inflammatory markers, or recent endoscopic or imaging evidence of disease activity. Second, in patients with back, neck, buttock, limb, or joint pain, clinicians should screen for inflammatory musculoskeletal features and consider rheumatologic evaluation for peripheral arthropathy, enthesitis, or axial spondyloarthritis. Third, patients with dermatomal pain, allodynia, burning pain, postsurgical pain, or complex regional pain syndrome-like features should be evaluated for neuropathic pain mechanisms. Fourth, patients with widespread pain, sleep disturbance, fatigue, mood symptoms, or pain disproportionate to inflammatory findings should be assessed for nociplastic or centrally amplified pain. Finally, patients with focal axial, radicular, myofascial, or peripheral nerve pain may benefit from pain-medicine evaluation for targeted rehabilitation, medication optimization, or selected interventional procedures. This approach emphasizes mechanism-based triage rather than assuming that persistent pain in IBD is synonymous with uncontrolled bowel inflammation.

The main strength of this study is the large real-world pain-clinic cohort spanning more than 15 years, with aggregation of nearly 20,000 clinical entries into patient-level and visit-level variables. The main limitations are the retrospective design and reliance on clinical documentation. The dataset did not contain complete demographic information, standardized pain scores, validated IBD activity indices, inflammatory biomarkers, endoscopic activity, disease duration, prior abdominal surgery, biologic exposure, rheumatologic diagnoses, psychiatric comorbidities, imaging findings, or longitudinal treatment-response measures. Therefore, adjusted analyses for age, sex, rheumatologic disease, inflammatory activity, and structural spine disease could not be performed. The study also did not include a matched non-IBD pain-clinic control group or a denominator of all IBD patients treated at the institution. Documentation frequency may reflect clinical complexity and follow-up duration as much as true symptom burden. Medication variables were based on chart documentation rather than complete pharmacy records. Finally, because the cohort was ascertained through pain-clinic encounters at a tertiary care medical center, it cannot be generalized to all patients with IBD.

**Figure 4:**
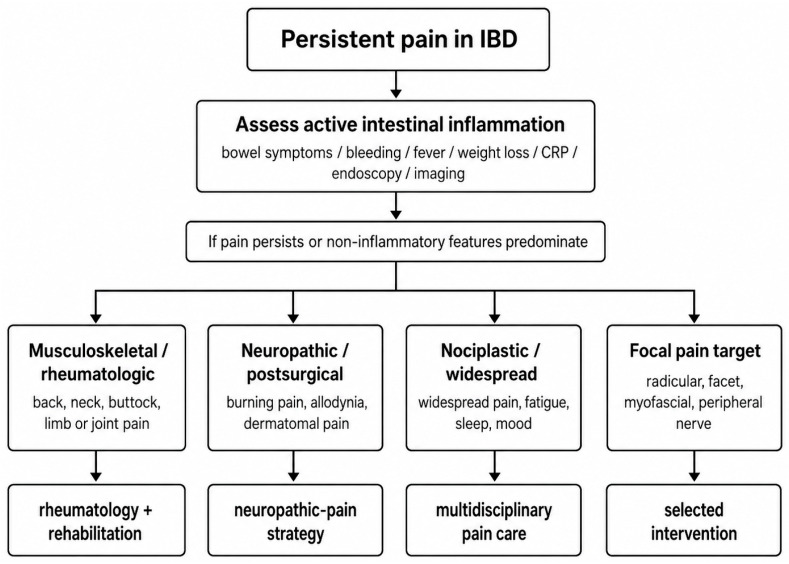
Proposed mechanism-based referral clinical framework for persistent pain in IBD. Flow diagram beginning with assessment of active intestinal inflammation, followed by screening for musculoskeletal/rheumatologic pain, neuropathic pain, nociplastic or widespread pain, and focal interventional pain targets.

Despite these limitations, the study defines a clinically important referral phenotype. Among patients with IBD who reach tertiary pain care, the dominant picture is not isolated abdominal or pelvic pain. Instead, these patients demonstrate a mixed pain phenotype with high rates of musculoskeletal, axial, neuropathic, widespread, and procedurally treated pain. Prospective studies incorporating standardized pain instruments, validated IBD disease-activity indices, inflammatory biomarkers, rheumatologic assessment, matched control groups, and longitudinal treatment outcomes are warranted (Figure 4).

### Future Directions

Future studies should prospectively validate these findings in multicenter IBD cohorts that include both a general IBD denominator and matched non-IBD pain-clinic controls. Such studies should collect demographic variables, IBD duration and phenotype, prior abdominal and perianal surgery, biologic and small-molecule exposure, opioid exposure, inflammatory biomarkers, endoscopic or radiologic activity, rheumatologic diagnoses, spine imaging, psychiatric comorbidity, and socioeconomic variables. Standardized pain instruments, neuropathic pain scales, fibromyalgia or nociplastic pain screening, quality-of-life measures, and procedure-specific pre- and post-treatment outcomes should be incorporated. Future work should also test whether mechanism-based triage algorithms can reduce unnecessary escalation of anti-inflammatory therapy, improve selection for rheumatology or pain-medicine referral, and identify patients most likely to benefit from neuromodulators, rehabilitation, behavioral interventions, or selected interventional pain procedures.

## 5. Conclusions

In this retrospective tertiary pain-clinic cohort of 596 patients with IBD, pain presentations were heterogeneous and were dominated by limb or peripheral joint pain, back or axial spine pain, abdominal or pelvic pain, neuropathic or CRPS-like features, neck pain, and widespread pain rather than by visceral abdominal pain alone. Interventional pain-care exposure was common, with 73.3% of patients having documentation of at least one interventional procedure. Crohn’s disease was associated with higher documentation of abdominal or pelvic pain and fibromyalgia-like or widespread pain, whereas ulcerative colitis was associated with greater documentation of selected spine-directed procedures. Because these comparisons were unadjusted and the dataset lacked demographic, inflammatory activity, imaging, rheumatologic, and treatment-response variables, the findings should be interpreted as descriptive and hypothesis-generating. Overall, the study supports a structured, mechanism-based approach to persistent pain in IBD, integrating gastroenterology, rheumatology, and pain medicine rather than assuming that persistent pain reflects uncontrolled intestinal inflammation alone.

## Figures and Tables

**Table 1:** Cohort construction and referral-context interpretation.

Measure	Value	Interpretation
Source population	IBD patients evaluated in pain-clinic settings	Specialty referral cohort; not a general IBD denominator
Clinical entries	19,615	Repeated entries aggregated into visits and patient-level variables
Unique pain-clinic visits	7053	Unique patient–medical record–date combinations
Unique patients	596	Unit of primary patient-level analysis
Visit date range	24 October 2010–14 May 2026	More than 15 years of clinical documentation
Mean visits per patient	11.8	Skewed by high-utilization patients
Median visits per patient	4	IQR, 1–11
Maximum visits for one patient	415	Indicates a small but extreme high-utilization subgroup
Patients with ≥2 visits	434/596	72.8%
Patients with ≥5 visits	264/596	44.3%
Patients with ≥10 visits	165/596	27.7%
Primary caveat	Pain-clinic referral cohort	Do not interpret pain frequency as population prevalence in IBD

**Table 2:** IBD diagnosis distribution.

Diagnosis	Patients, n/N (%)	Unique Visits, n
Crohn’s disease	314/596 (52.7%)	3606
Ulcerative colitis	247/596 (41.4%)	2947
IBD-unclassified/other	35/596 (5.9%)	500
Total	596/596 (100%)	7053

**Table 3:** Patient-level pain phenotypes.

Pain Phenotype	n/N	Percent
Limb/peripheral joint pain	395/596	66.3%
Back/axial spine pain	358/596	60.1%
Abdominal/pelvic pain	246/596	41.3%
Neuropathic or CRPS-like features	198/596	33.2%
Neck pain	188/596	31.5%
Head/craniofacial pain	147/596	24.7%
Fibromyalgia-like/widespread pain	130/596	21.8%

**Table 4:** Visit-level pain phenotype documentation.

Pain Phenotype	Percent of 7053 Visits
Back/axial spine pain	45.9%
Limb/peripheral joint pain	44.3%
Abdominal/pelvic pain	22.0%
Neck pain	15.2%
Neuropathic or CRPS-like features	13.5%
Fibromyalgia-like/widespread pain	12.1%
Head/craniofacial pain	10.8%

**Table 5:** Interventional pain procedures.

Procedure Category	n/N	Percent
Any interventional procedure	437/596	73.3%
Epidural or nerve-root block	181/596	30.4%
Facet or medial branch procedure	171/596	28.7%
Radiofrequency procedure	145/596	24.3%
Trigger-point injection	76/596	12.8%
Occipital nerve block	50/596	8.4%
Sympathetic block	12/596	2.0%

**Table 6:** Medication and treatment-class documentation.

Medication/Treatment Class	n/N	Percent
Gabapentinoid documentation	176/596	29.5%
SNRI/TCA documentation	146/596	24.5%
Opioid documentation	68/596	11.4%
Systemic steroid documentation	231/596	38.8%

**Table 7:** Selected Crohn’s disease versus ulcerative colitis comparisons.

Outcome	Crohn’s Disease, n/N (%)	Ulcerative Colitis, n/N (%)	Odds Ratio (95% CI)	*p*-Value
Abdominal/pelvic pain	148/314 (47.1%)	82/247 (33.2%)	1.79 (1.27–2.53)	0.001
Fibromyalgia-like/widespread pain	83/314 (26.4%)	39/247 (15.8%)	1.92 (1.25–2.93)	0.0027
Radiofrequency procedure	59/314 (18.8%)	78/247 (31.6%)	0.50 (0.34–0.74)	0.0005
Facet or medial branch procedure	79/314 (25.2%)	87/247 (35.2%)	0.62 (0.43–0.89)	0.012

Odds ratios are expressed for Crohn’s disease relative to ulcerative colitis. These comparisons are exploratory and unadjusted.

## Data Availability

Deidentified aggregate data supporting the findings are available from the corresponding author upon reasonable request, subject to institutional and regulatory approvals.
